# The effect of posterior and lateral approach on patient-reported outcome measures and physical function in patients with osteoarthritis, undergoing total hip replacement: a randomised controlled trial protocol

**DOI:** 10.1186/1471-2474-15-354

**Published:** 2014-10-27

**Authors:** Signe Rosenlund, Leif Broeng, Carsten Jensen, Anders Holsgaard-Larsen, Søren Overgaard

**Affiliations:** Department of Orthopedic Surgery and Traumatology, Odense University Hospital, Institute of Clinical Research, University of Southern Denmark, Odense, Denmark; Department of Orthopedic Surgery and Traumatology, Køge Hospital, Køge, Denmark

**Keywords:** Osteoarthritis, Hip replacement, Surgical approach

## Abstract

**Background:**

Total hip replacement provides pain relief and improves physical function and quality of life in patients with end-stage hip osteoarthritis. The incidence of hip replacement operations is expected to increase due to the growing elderly population. Overall, the posterior approach and lateral approach are the two most commonly used approaches for hip replacement operations. The posterior approach is associated with an increased risk of revision due to dislocations, and some studies have shown that the lateral approach is associated with reduced patient-reported outcomes, including physical function and pain; however, this has not been investigated in a randomised controlled trial with a twelve-month follow-up. We hypothesized that the lateral approach has an inferior outcome in patient-reported outcome compared with the posterior approach after one year.

**Methods/Design:**

The trial is a prospective, double blinded, parallel-group controlled trial with balanced randomisation [1: 1]. Patients with hip osteoarthritis scheduled for hip replacement surgery, aged 45–70 years, will be consecutively recruited and randomised into two groups. Group A will receive hip replacement using the posterior approach, and Group B will receive hip replacement using the lateral approach. The primary end-point for assessing the outcome of the two interventions will be twelve months after surgery. Follow-up will also be performed after three and six months. The primary outcome is Hip Disability and Osteoarthritis Outcome Score, subscale of "Physical function Short form" (HOOS-PS) Secondary outcome measures include two other subscales of HOOS ("Pain" and "Hip related Quality of Life"), physical activity level (UCLA activity score), limping (HHS) and general health status (EQ-5D-3L). Explorative outcomes include physical function test, 3D-gait-analysis and muscle strength.

**Discussion:**

To our knowledge, this is the first randomised controlled trial comparing the posterior approach with the lateral approach with patient reported outcome as the primary outcome and with a twelve-month follow-up.

**Trial registration:**

Clinicaltrial.gov:NCT01616667.

**Electronic supplementary material:**

The online version of this article (doi:10.1186/1471-2474-15-354) contains supplementary material, which is available to authorized users.

## Background

Hip osteoarthritis (OA) is a common and progressive joint disease causing pain, reduced physical function and reduced quality of life. Total hip replacement (THR) provides pain reduction and improves physical function and quality of life in most patients with end-stage hip OA[1, 2]. In the US, 427,000 hip replacements are performed each year[3]. In Denmark (DK), approximately 10,000 primary and 1,600 revision operations are performed each year, and the incidence is expected to increase due to the growing elderly population[4]. In DK, the majority (95%) of the procedures are performed using the posterior approach (PA). In contrast, the lateral approach (LA) is more widely used internationally[5, 6], and overall PA and LA are the two most commonly used approaches for THR[6, 7].

PA is associated with an increased dislocation rate and revision rate due to dislocations compared with LA[5, 8, 9]. This may be due to dysfunction of the posterior soft tissue structures after PA surgery, although these are repaired in some cases[10]. Regarding patient-reported outcome measures (PROM), some studies comparing PA with LA have shown that LA is associated with reduced outcome, including physical function and pain[6, 11, 12]. Furthermore pronounced limping[13, 14] and reduced hip abductor muscle strength may be associated with LA[15, 16]. This might be explained by the surgical damage on the lateral structures around the hip joint[6, 7, 17]; however, these outcomes have never been evaluated in randomised controlled trials (RCT) with more than three month follow-up[18]. A Cochrane review from 2004 did not include PROMs as outcome measures due to lack of studies evaluating the patient perspective[7]. Thus, the extent to which the choice of approach affects the outcome from a patient’s perspective is largely unknown[7].

To investigate the influence of surgical approach on patient-reported outcome after THR and with the perspective of reducing risk of revision, an RCT is needed that investigates potential differences between PA and LA. This trial will provide new evidence regarding the patient’s perspective, including a twelve-month follow-up, upon which the choice of approach can be made.

### Aim

The primary aim of this trial is to evaluate the postoperative effect of surgical approach after THR on patient-reported physical function. The secondary aim is to evaluate the effects on patient-reported pain, physical activity and quality of life; further, to evaluate objective measures of physical function, gait and hip muscle strength. We hypothesize that patient-reported and objective outcome measures within the first year will improve more in patients receiving the PA compared with LA.

## Methods/Design

### Study design

A prospective, double-blinded, parallel-group controlled trial with balanced randomisation [1:1], in accordance with CONSORT guidelines[19]. The trial is registered at ClinicalTrial.gov (NCT 01616667).

### Participants and recruitment procedure

Patients will be referred from general medical practitioners to the outpatient clinic at the Department of Orthopaedic Surgery and Traumatology, Odense University Hospital (OUH). The clinic has two locations; one in the city of Odense at OUH, and one in the city of Svendborg at Svendborg Hospital, Denmark. Patients, aged 45–70 years, with indication for cementless THR based on symptoms, clinical and radiological findings, will be screened according to the in- and exclusion-criteria, listed in Table 1. Eligible patients will be given oral and written information about the trial. Subsequently, an appointment with the principal investigator (SR) is made, and the patients are screened again according to the exclusion criteria. The 30 second chair-stand-test (30s-CST) and Orientation-Memory-Concentration Test (OMC test) will be performed to ensure that no patients have severe medical conditions compromising their physical performance or any mentally disturbances influencing their ability to cooperate during physical testing or complete questionnaires. The patients will be given an introduction to completing a patient diary and filling in the questionnaires. Upon signing the informed consent, the baseline measures are performed. Finally, patients will be randomised and scheduled for operation by a secretary not involved in the trial.

**Table 1:** **Criteria for participants in the trial**

Inclusion criteria	Exclusion criteria
Age: 45–70 years, both years included	More joints (hip, knee or ankle) with expected joint replacement within a year
Patients scheduled for primary cementless total hip replacement	Prior joint replacement on any joints (hip, knee or ankle), or any joint related surgery on lower limbs, still providing symptoms
Endstage primary hip OA or secondary OA due to mild hip dysplasia (CE-angle >20 degrees)	BMI >35
	Any physical disability preventing patients from walking 20 meters without aid
	Any neurological disease (ex. cerebral thrombosis, Parkinson) compromising the walking ability
	Any severe medical condition compromising the physical function (ex. Chronic heart failure, chronic obstructive pulmonary disease) Evaluated by 30s-CST-test
	Severe dementia (OMC < 18)
	Inability to read and understand Danish writing and oral instructions
	Does not wish to participate

### Setting

Baseline measurement, randomisation, THR operation and follow-up will take place at OUH, Odense. The surgical intervention will be performed by a surgeon from one of two teams of experienced orthopaedic specialists. One team consists of three surgeons, all with special training in LA, and they will perform all LA operations. The other team consists of three surgeons, all with special training in PA, and they will perform all PA operations. Selection of component sizes will be based on preoperative templating performed in TraumeCad®, using standardised pelvic and anterior-posterior hip x-rays and finally adjustments on findings during surgery, if necessary. All patients will receive the same type of cementless components (Bi-metric stem® and Exceed ABT Ringloc-x Shell™). Standardised information about surgical procedure, complications, care during hospital stay and rehabilitation will be given. All patients will receive a standardised medical regime, including pre- and post- operative antibiotic within the first 24 hours, and 7 days of thrombo-prophylaxis. Local analgesia infiltration (150 ml Naropin® (2 mg/ml), 1 ml Toradol® (30 mg/ml) and 0.5 ml Adrenalin (1 mg/ml)) will be given before closure of the wound. Postoperatively, the patients will be given a standardised self-administrated analgesic regime that includes paracetamol 1000 mg four times daily and 10 mg morphine tablets when needed. If any additional or alternative analgesic treatment is necessary, this will be recorded in the patient dairy. The patients will be instructed on how to take the morphine tablets by the principal investigator (SR) prior to the operation and by ward nurses during the in-hospital stay. They are instructed to take a tablet if they have pain >3 on the numeric ranking scale (NRS) for pain (see Explorative Outcomes), when at rest in bed/chair, or when they are in pain ≥5 on the NRS scale during activity. The patients will each day receive a random number (six to eight) of morphine tablets in a small plastic bag. If the patient needs more tablets, than are in the bag, the ward nurses will provide the patient with additional tablets, and this will be registered. After 24 hours, the bags will be recollected and remaining tablets will be counted and registered. The postoperative mobilisation will be performed by a physiotherapist. Standardised rehabilitation protocol (no matter which type of intervention) will be applied, including weight bearing as tolerated and no movement restrictions. The patients will be mobilised to standing position on the day of operation. The following days, the patients will be trained in transfer situations needed in daily activities (in and out of bed, up and down from chair) and walking with two canes and on stairs. Following surgery the patients are instructed in a home based rehabilitation program with 11 exercises. The 11 exercises’ primary focus is strengthening of the hip muscles by use of elastic bands. The patients are instructed to conduct this program three times a day, repeating each exercise 10 times for each leg. When they can perform 30 repetitions of each exercise on the operated leg, they should increase the intensity by using an elastic band around the ankles during exercise. They are instructed to continue the exercise program for at least three months. If the physiotherapist finds it necessary, the patient will be referred to additional training provided by a local physiotherapist. The latter will be recorded.

### Intervention

During the operation, all patients will be positioned in lateral decubitus position.

#### Posterior approach

PA is performed through an incision over the posterior part of greater trochanter through the fascia, followed by blunt dissection of gluteus maximus. Then detachment of the external rotators and incision of the posterior part of the hip capsule[20]. The hip is dislocated by internal rotation and flexion. During closure, capsular repair and re-insertion of the external rotators are performed.

#### Lateral approach

LA is performed through a midline incision over the greater trochanter and involves detachment of the anterior one-third of the gluteus medius insertion and gluteus minimus insertion on the tip of greater trochanter. Excision of the hip capsule is performed on the anterior side of the joint, from the basis of collum femoris to the acetabular rim. The hip is dislocated by external rotation, adduction and flexion. During closure of the wound, re-insertion of the detached part of muscle gluteus medius and muscle gluteus minimus is performed. There is no capsular repair[14]. All wounds will be closed with nylon to avoid visible suture clips on the postoperative radiographs.

### Outcome measures

One primary outcome has been chosen to avoid problems with interpretations associated with multiplicity of analysis[19]. The primary outcome will be supported by several other patient reported secondary and tertiary outcome measures, further explored by a range of explorative outcome measures.

### Primary outcome

#### Physical function

Hip Disability and Osteoarthritis Outcome Score (HOOS 2.0), subscale "Physical Function Short form (HOOS-PS)", will be used as the primary outcome, with primary endpoint after twelve months. The subscale HOOS-PS is an aggregation and shortening of the two original subscales of HOOS-ADL and Sport and Recreation. It has been developed to optimize the measurement of physical function and at the same time lower the burden of long questionnaires for the patients. The HOOS-PS subscale includes five items (three from HOOS-ADL and two from HOOS sport and recreation) that cover a wide range of physical functions, from low demand to high demand functions[21] It is a disease-specific patient-reported outcome measure developed to assess the patients’ opinion about their hip associated problems[22–24]. The subscale ranges from 0 point (extreme symptoms) to 100 point (no symptoms). HOOS-PS has been evaluated regarding validity and responsiveness in THR patients. HOOS-PS was found to have high internal consistency and responsiveness[24]. HOOS 2.0 has been evaluated regarding validity and reliability in THR patients. It was found to have high content validity, construct validity, test-retest reliability, responsiveness and interpretability[22]. One study also found good internal consistency and little floor and ceiling effect[25]. All items of HOOS 2.0 has been translated into Danish[26].

### Secondary outcomes

#### HOOS 2.0

HOOS subscale for pain, and hip related Quality of life (QoL) will also be reported.

#### General health status

EuroQol/*EQ*-*5D*[27] is a patient-reported generic general health questionnaire. The first part evaluates the following five dimensions; mobility, self-care, normal activities, pain/discomfort and anxiety/depression, and uses a 3-point Likert scale for each dimension. The second part evaluates the patients’ perception of their overall health and is scored on a 100-point visual analogue scale. EQ-5D has been validated in knee OA patients[28], where the construct validity was found acceptable. Although EQ-5D is not validated in a THR population, it can be recommended for evaluating health related quality of life (HRQoL) with EQ-5D in these patients[29]. The test-retest reliability has been shown moderate to good in healthy and knee OA patients[28, 30]. The responsiveness was good in rheumatoid arthritis populations and in patients with femoral neck fractures[31, 32]. The floor effect is negligible, but a high ceiling effect has been found in THR patients[33]. *EQ*-*5D* is translated into Danish[34].

#### Physical activity

The University of California Los Angeles activity score (UCLA) uses a 10-point Likert scale to evaluate activities, ranging from inactive to regular participation in impact sport or heavy labour. UCLA has been validated in patients undergoing hip or knee joint replacement[35] with good construct validity, test-retest reliability[35, 36], moderate responsiveness, low ceiling and floor effect[36]. UCLA contributes with important qualitative information on the patients’ physical activity in correlation with other clinical outcome measures[35, 37]. UCLA is translated into Danish.

#### Limping

Harris Hip Score (HHS) is a disease-specific surgeon-reported questionnaire. It includes four domains: pain, function (subdivided in ADL and gait), deformity and joint motion. In this trial, only the question from the function domain, regarding the amount of limping, is used. It is scored on a 5-point Likert scale (none, slight, moderate, severe limp or unable to walk). The question will be used as a patient-reported outcome to evaluate the patient’s own experience of limping during gait. The reliability and validity of HHS is tested in a THR population and showed low floor effect but high ceiling effect. The test-retest and inter-observer reliability was good. Internal consistency showed high Cornbach α value in each domain[38]. HHS is translated into Danish.

### Explorative outcomes

#### Physical function test

Prior to each test, the patient will be orally instructed on how to perform the test. Regarding all maximal physical function tests, the patient is instructed to perform the test as fast as possible; however, while still feeling secure. No oral feedback will be provided during the tests. Assistive devices will be allowed at the follow-up assessments when needed, but not at baseline, according to the exclusion criteria. However, use of the arms to assist rise from the chair in 30s-CST is allowed. Both will be recorded.

#### The 20 meter walk-test

In the 20 meter walk-test (20WT), patients are instructed to walk 20 meters between two clearly visible lines marked on the floor. The time for both self-selected normal and maximal pace is measured, and the number of steps is counted. The stopwatch is started on the command "go" after a countdown from "ready, set, go". The mean velocity of two trials will be used for further analysis. Good agreement and excellent test-retest reliability have been demonstrated in both hip and knee OA patients[39].

#### "Timed Up and Go" test

The "Timed Up and Go" test (TUG) measures the time it takes a person to rise from a chair (seat height 44 cm, with armrests), walk three meters to a clearly visible line marked on the floor, turn and walk back to the chair and sit down again. The stopwatch is started on the command "go" after a countdown from "ready, set, go". The best out of two trials will be used for further analysis[40]. TUG is used for quantifying functional mobility. Good construct validity has been reported in knee replacement patients[41, 42]. Moderate to good test-retest and inter-rater reliability have been shown in patients with hip and knee OA[43–45].

#### The 30 sec-chair-stand-test

The 30 sec-Chair-Stand-test (30s-CST) measures the number of "stands" completed (seat height 44 cm, with armrests) within 30 seconds[46]. The best out of two trials will be used for further analysis. The stopwatch is started on the command "go" after a countdown from "ready, set, go". The test assesses the overall strength of the lower limb muscles. The 30s-CST has showed valid as a measure for lower limb muscle strength in active older adults (60+)[46], and the test showed good intra- and inter-rater reliability in patients with moderate to end-stage hip or knee OA[44, 45, 47].

#### The 30 sec repeated unilateral knee bending test

The 30 sec repeated unilateral knee bending test (30s-knee bend) evaluates the ability to execute fast coupled eccentric-concentric muscle force, with a primary focus on knee muscle function and a secondary focus on hip muscle function. The maximum number of knee bends completed on one leg within 30 seconds will be recorded. The patient stands aligned with the front of their foot touching a straight line taped to the floor; fingertip support for balance is provided by the examiner. The patient is then asked to bend his/her knee, without forward trunk lean, until he/she is not able to see the line at their toes (about 30° of knee flexion)[48]. Three to four knee bends are performed on each leg to familiarise the patient with the depth of the flexion. The unaffected leg is tested first. The stopwatch is started on the command "go" after a countdown from "ready, set, go". If the opposite (raised) leg touches the ground, the trial is stopped, and the number performed is recorded. One trial on each leg will be performed. The test was found valid to discriminate between symptomatic and non-symptomatic leg in meniscectomised patients[48]. Also, it has showed low floor and ceiling effect in meniscectomised patients[48]. It was found reliable with moderate agreement and good test-retest reliability in patients with hip and knee OA[39].

#### Trendelenburgs test

The Trendelenburg test will be performed according to Hardcastle et al.[49]. It indirectly measures the hip abductor muscles strength, but the validity is disputed[50, 51].

#### Hip ROM

Passive hip range of motion (ROM) will be assessed with the patient in the supine position, and prone when assessing hip extension. Hip ROM is defined as the range of movement that an examiner is able to move the hip joint through its full range with no active participation from the patient[52]. Hip ROM in flexion/extension, abduction/adduction and internal/external rotation will be measured in degrees using a standard hand-held goniometer (30 cm)[53]. The reproducibility has been evaluated in patients with mild to moderate hip OA and was found poor[53].

### Gait analysis and hip muscle strength

#### Gait deviation index

Gait Deviation Index (GDI) is calculated from kinematic data collected from 3D-gait-analysis during horizontal gait. The GDI expresses the degree of gait pathology in patients compared to healthy subjects, with a mean GDI of 100 point[54]. 3D gait-analysis is extensively used to objectively collect both temporospatial, kinematic and kinetic data. GDI can simplify the complexity of the kinematic gait data in THR and other populations[17, 55, 56]. In unpublished data from our own gait laboratory, we found good test-retest reliability on GDI. GDI has been validated in children with cerebral palsy[57].

#### Hip muscle strength

Isometric maximal voluntary muscle contraction (MVC) will be used to assess the maximal muscle strength of the hip abduction, hip flexion and hip extension. MVC is collected standing, according to the protocol described by Jensen C. et al.[58]. MVC and rate of force development (RFD) will be analysed for both legs. A simple dice randomisation will be used to determine starting leg and the sequence of MVC exercise, avoiding a systematic leg-to-leg learning bias.

For each muscle group, three test contractions will be performed. The contraction with peak MVC will be selected for further analysis. The patient will receive both visual feedback on a monitor and verbal feedback during each test. MVC has been found reliable in THR patients[58].

#### EMG

Surface electromyography (sEMG) is the myoelectric signals associated with a contraction of a muscle, measured with a pair of surface electrodes on each muscle. We will use the SENIAM guidelines[59] for skin preparation and electrodes placement on five selected lower extremity muscles: muscle gluteus medius, muscle tensor facia lata, muscle gluteus maximus, muscle rectus femoris, and muscle semitendinosus. EMG will be collected both during gait and MVC measurement[55, 60]. Peak and mean EMG signal- (absolute/normalised to MVC) and integrated EMG during stance phase will be analysed. The test-retest reliability of sEMG from muscle gluteus medius normalised to MVC has been evaluated in one study on 13 young healthy persons, and good reliability was found. Also, the intra- and inter-individual variability of EMG during gait, normalised to MVC in young healthy persons, has been found good and is therefore recommended[61, 62].

To ensure inter-examiner uniformity, a detailed written laboratory protocol describing the procedure and the oral instructions given to the patients during the physical function tests and MVC assessment has been developed. The protocol has been rehearsed before evaluating the study participants.

### Patient diary

The patients will complete a diary consisting of four parts; 1) Pain at rest is measured four times daily using an 11-point box Numeric Ranking Scale (NRS). The NRS has been found a reliable, valid and a responsive tool in geriatric patients[63]. 2) Consumption of painkillers. Type, dosage and frequency of pain killers will be recorded. This is recommended in recent evidence-based guidelines for management of hip and knee OA[64] 3) The need for continuous cane use and 4) Completing the HOOS-pain questionnaire. The diary will be filled in each day for the first five days and then every second week until 3 months after surgery.

Digital scanning will be used for questionnaires, and manual double data entry will be done[65].

### Assessments and follow-up period

Assessments point is presented in Table 2. A reminder will be sent per SMS on all the selected days for follow-up. The patients will receive the first set of questionnaires at the outpatient clinic at baseline. The following three sets, including return envelopes, will be handed out during the in-hospital stay. Furthermore, a reminder will be send per SMS at three, six and twelve months. In case of missing return, a new set and a reminder to complete the questionnaires will be sent by ordinary post service. All questionnaires will be completed at the patients’ home. In case of missing visits for physical function test or assessment in the gait laboratory, the patient will be contacted by phone, and a new appointment will be scheduled.

**Table 2:** **Outcomes and assessment points**

		Assessments point			
Primary outcome	Data collection instrument (unit)	Baseline	Post (days)	Post (weeks)	Post (months)
Function of daily living	HOOS-PS	Pre			3, 6, 12
Secondary outcomes					
Patient reported outcomes					
Pain	HOOS-pain	Pre			3, 6, 12
Quality of life	HOOS-QoL	Pre			3, 6, 12
Physical activity	UCLA	Pre			3, 6, 12
General health	EQ-5D	Pre			3, 6, 12
Limping	Harris Hip Score	Pre			3, 6, 12
Explorative outcomes					
Pain					
Pain	HOOS-pain	Pre	5	2- 10	3
Use of medication	(type, quantity)	Pre	1- 5	2- 10	3
Short term pain measure	NRS	Before and after each test (pre- 12 months)			
Physical function					
20 Meter Walk test (20WT)	Stopwatch (s and steps)	Pre	3	3	3
Timed Up and Go test (TUG)	Stopwatch (s)	Pre	3	3	3
Chair Stand - test (30s-CST)	(quantity)	Pre	3	3	3
Knee Bend test (30s-knee bend)	(quantity)	Pre	3	3	3
Trendelenburg test	(positive/negative)	Pre			3
Joint mobility (Hip ROM)	Goniometer (degrees)	Pre			3
Data from 3D-gait analysis					
Total score for kinematic data	GDI (points)	Pre			3, 12
EMG	(frequency and amplitude)	Pre			3, 12
Hip Muscle strength	MVC (Nm/kg)	Pre			3, 12
Rate of forced development	0-200 ms MVC (Nm)	Pre			3, 12

### Adverse events

A medical record audit will be performed at twelve months post-surgery. Adverse events regarding periprosthetic fracture, nerve palsy, wound infection, deep thrombosis or pulmonary embolism, dislocation and revisions will be collected. Pain will be assessed before and after all sessions of physical function test, gait-analysis and MVC measurements. Pain >5 on the NRS is considered an event that may influence the patients’ effort of maximal performance. The assessment of pain will provide information about the feasibility of the test battery. No interim analysis or stopping guidelines are planned. Both interventions are well known and routinely performed with few severe adverse effects. However; we will register and report any adverse events to the appropriate health authorities.

### Sample size

Sample size calculation was performed upon the primary outcome HOOS-PS, using one preoperative and three follow-up assessments and an estimated correlation between follow-up measurements of 0.5. We considered a minimal important difference of 10 points between the two groups at 12 months follow-up to be of clinical relevance[66–69] and used a standard deviation of 16.7 and 16.1 postoperatively[24]. We have performed our sample size calculation using the function "sampsi method(change)" supported by the Stata 13 software and described by Frison and Pocook[70]. To achieve a statistical power of 80% (β = 0.80), it was calculated that a sample size of n = 29 was needed in each intervention group in order to detect statistical significant differences at α = 0.05 level. N = 40 was used to account for possible drop-outs. A secondary sample size estimation was conducted to estimate the number of patients needed for analysing the primary explorative GDI. We had á prior defined that a clinically relevant improvement in gait function measured in GDI is 7.5 point, which corresponds to half a standard deviation in the only available randomised clinical trial using GDI as outcome measure[71]. From data collected in our own gait laboratory we know that the standard deviation in hip OA-patients is 9 point preoperatively[72]. To achieve a statistical power of 80% (β = 0.80), it was calculated that a sample size of n = 17 was needed in each intervention group in order to detect statistical significant differences at α =0.05 level. Sample size in each group on n = 20 was used to account for possible drop-outs.

### Randomisation

**Figure 1:**
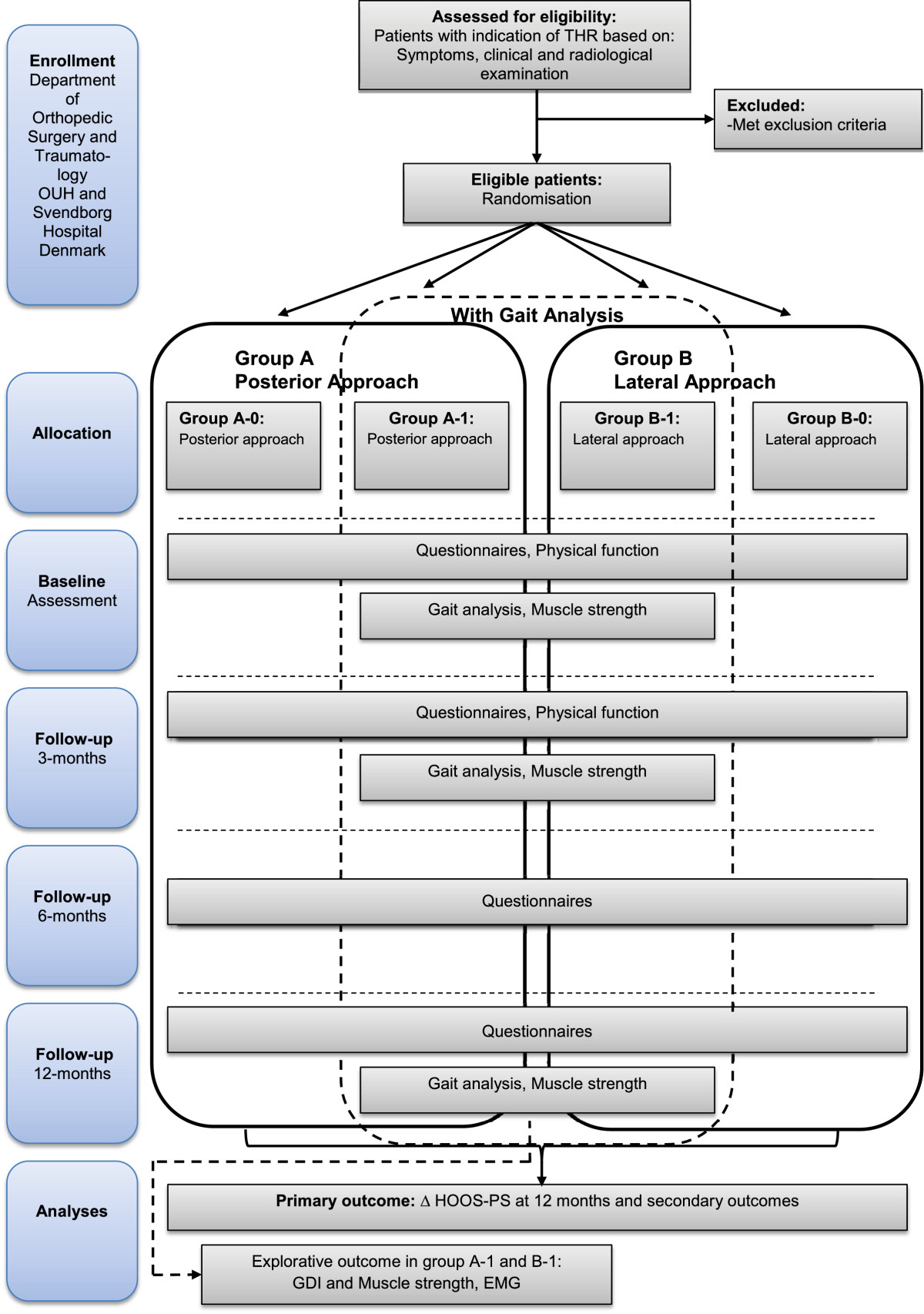
**Study flow chart.**

Balanced 1:1 block randomisation was performed using a computer-generated list containing a sequence of one letter and one number; "A" referring the patient to posterior approach, "B" referring the patient to lateral approach. "1" referring the patient to participate in the gait analysis and thus contributing with data on GDI, and "0" referring the patient not to participate in the gait analysis (Figure 1). Four blocks of 20 patients each was generated. The sequence was generated by a third person (JL) not involved in the trial. The letter and number combination was written on paper, folded and placed in sealed opaque consecutively numbered envelopes. The booking secretary will open the envelopes in the given order, and according to the content the patient will be scheduled for operation. In the first three blocks there will be a 66% chance of being allocated to group 1(gait analysis). This will enable us to verify the sample size calculation of 2x20 patients for the GDI. A recompilation will be made, based on ungrouped results of GDI for the 20 first actual gait analyses performed. From this, we will adjust the final number of patients allocated to gait analysis accordingly.

### Blinding

The patients will be blinded to treatment. The patients are told prior to participation that they will not be informed about the type of intervention and they are carefully explained the reasons for this. Also the health care providers (doctors, nurses and physiotherapists) are all explained the importance of not talking about the intervention with the patients, and since the patients post operatively are treated after the same rehabilitations protocol, there is no need to discuss interventions. Blinding to treatment allocation (surgeons, ward nurses and, physiotherapists) is not possible due to the nature of the intervention. However, the data collectors (except one (DBN) performing gait analysis) and the principal investigator (SR) analysing the data will be blinded throughout the trial. We will use recoded identification numbers when analysing the data. The recoding will be performed by an independent person (JL).

### Statistical analysis

Baseline characteristic of study participants, will be presented with mean and SD, for all relevant outcomes, according to CONSORT item 15[19]. The primary statistical analysis will be performed between the two groups on mean change in HOOS-PS from baseline to twelve months follow-up. To evaluate the treatment effect (mean difference between the groups at three, six and twelve months), we will employ a random effect mixed linear model analysis (repeated measures) with point estimates[73]. This model includes the interaction between treatment and elapsed time, adjusted for baseline values and assuming that data is missing completely at random (MCAR)[73]. Model assumptions will be checked by residual plots. The secondary statistical analysis will include the same approach as described above for all the secondary outcomes. We will present the results with mean and standard deviation for both baseline and follow-up measurements in the two groups. Furthermore we will present the adjusted difference in mean between the groups at 12 months including 95% CI. Finally, the effect size using Cohen’s *d* effect size with indexes for small, medium and large effect as proposed by Cohen, 1992 will be reported[74]. The intention-to-treat (ITT) principle will be applied[75]. Subsequent per-protocol analysis may be needed in case a substantial number of data is lost during follow-up. All data will be checked for Gaussian distribution, and parametric statistics will be used were appropriate. An α-level of 0.05 will be used, and data presented as means with 95 CI, unless otherwise stated. Finally, the number needed to treat (NNT) for a positive effect of treatment (>10 points on HOOS-PS) will be analysed. All statistical analyses will be blinded and performed using Stata 13 software (StataCorp, Texas, USA).

### Ethics

The trial complies with the Declaration of Helsinki. It is approved by the Danish Data Protection Agency and The Danish Regional Committee on Biomedical Research Ethics (Southern Denmark), Project-ID S-20120009. A written and orally informed consent will be collected prior to inclusion of all participants.

## Discussion

The choice of surgical approach and the influence on patient-reported outcome, physical function, hip muscle function and gait are often debated. Especially, the lateral approach and its impact on the hip abductor muscles are a concern that may be associated with decreased PROM, reduced physical function and increased limping. Interest in reducing the surgical induced muscle damaged is also emphasised by a study showing that decreased lower limb muscle strength is associated with deterioration of ADL functions[76]. Despite this, only few studies have evaluated PROM, physical function and gait between the PA group and LA group.

### PROM

The trial focuses on HOOS-PS as primary outcome after twelve months, along with a number of secondary outcomes, in order to evaluate the patients’ perspective on physical function (HHS-limping), physical activity (UCLA), pain (HOOS-Pain, EQ-5D) and quality of life (HOOS-QoL, EQ-5D). All of these are valid measurements and recommended for use in RCTs[77].

To our knowledge, only three studies have investigated PROM when comparing PA with LA[6, 11, 18]. Of these only one study is a randomised controlled trial (RCT) and did not use PROM as primary outcome. Furthermore, the three studies do not agree in their conclusions of which approach is superior in terms of patient reported physical function and pain. The two non-randomised cohort studies are demonstrating a small, but significant, difference in favour of PA. Contrary, the RCT did not find any differences in WOMAC, which was evaluated as a secondary outcome. Also, two studies have compared the PA with LA using Harris Hip Score (HHS)[12, 18]; however, HHS is not entirely patient-reported. The first study found that LA had a significant inferior outcome on total HHS after twelve months[12], whereas the other study found no differences after three months[18].

PROM is recommended by OMERACT (Outcome Measures in Rheumatologic Clinical Trials) as the core set of outcome measures in phase III clinical trials investigating OA treatment, including THR[77]. The core set outcome includes pain, physical function and patient global assessment. We have chosen the HOOS-PS as primary outcome for mainly three reasons. First, physical function has been defined by OMERACT as one out of three core set outcomes to evaluate. Physical function is well reflected in HOOS-PS, which includes 5 items. Second, we find it relevant to use HOOS-PS as primary outcome, because it reflects the possible negative influence of LA due to the surgical damage on the hip abductor muscles, which could lead to reduced physical function for these patients. Third, HOOS is in a recent review recommended for evaluation of THR patients, because it was found to be the best validated and a reliable PROM when compared with other commonly used PROMs (WOMAC, OHS, modified HHS etc.)[22].

Pain is also a core set outcome in the OMERACT guidelines[77]. Measurements of pain are represented in both the secondary and the explorative outcomes, by HOOS-pain, NRS and actual intake of painkillers. Lastly, a global patient assessment score is the third core set outcome in the OMERACT guidelines, and it will be measured by the patients’ perception of their overall health with the second part of EQ-5D. The use of a specific question regarding limping from HHS will provide important information on the gait quality perceived by the patients and will be further explored using GDI.

### Physical function

The surgical induced damaged to the abductor muscles (muscle gluteus medius and gluteus minimus) may contribute to a decrease of the physical function in the LA group[6, 7, 17]. Evaluating the overall early physical function and the gait performance will contribute with data that explore this hypothesis further. Also, the physical function test has shown greater responsiveness during the early stage after surgery than PROM[42], and it has been shown that the physical function test may capture a different construct of the patients function than patient-reported physical function. Patient-reported physical function has shown closer relation to the patients’ perception of pain[42]. For these reasons, the trial is designed with early measurement of the patients’ physical function, using a battery of valid and reliable tests.

### Gait and muscle strength

Several trials have found reduced maximal voluntary muscle strength (MVC) of the hip abductor muscles in the affected side amongst the THR population[58, 78–80]. However, only three non-randomised studies have investigated MVC of the abductor muscles comparing PA with LA. Gore et al.[15] found reduced abductor muscle strength in the LA group, while equal muscle strength between the two approaches was reported by Downing et al.[81] and Kiyama et al.[78]. Thus, no firm conclusion can be draw from the existing literature. Previously, hip abductor muscle weakness during gait, evaluated with 3D-gait analysis, has also been reported in the general THR population[56, 60, 79, 82]. However, only one study investigating the quality of gait using 3D-gait analysis has compared LA with PA. This study concluded that all the LA-patients demonstrated an abnormal gait pattern, while 30% of the PA-patients had a normal gait pattern six month after surgery[17]. It was suggested that the observed abnormal gait pattern in the LA-group was a compensatory mechanism potential caused by hip abductor weakness, even though muscle strength was not assessed[17]. A recent review investigating gait quality of the THR-population compared with healthy controls found impairment in several outcomes, including gait speed and hip abductor muscle function in the THR group[83]. Because only few studies until now have been conducted, Ewen et al. emphasise the importance of investigating the potential differences between surgical approaches in the future[83]. In summary, the quality and quantity of information extracted from studies comparing LA with PA performed to date are insufficient to draw any firm conclusions about physical function, muscle strength and gait.

### Study design

The internal validity of the trial is good, due to same recruitment procedure, same in/exclusion criteria, same team taking care of the patients, and the patients experience the same amount of extra attention regarding the additional outpatient visits related to testing. The age limits and the list of exclusion criteria can affect the external validity and generalizability. However; patients under the age of 45 are likely to suffer from secondary OA and in our department all patients over the age of 70 receive a standard treatment with a cemented prosthetic concept. Thus, to ensure high internal validity by avoiding subgroup analysis due to different prosthetic concepts, we decided only to include patients aged 45–70. The exclusion criteria facilitate investigation of the potential differences between the two approaches due to the impact on different soft tissue structures. Thus, the exclusion criteria enable the investigation of the isolated effect of the two approaches, without interference of any other health conditions, which is the primary aim of this trial. We have chosen the primary end-point at twelve months after operation, as we expect a stable rehabilitations level at this point[42]. The intermediate assessments at three and six months will provide information about potential differences in the progression of rehabilitation between the two groups.

We have chosen block randomisation for two reasons: 1) because logistic challenges exist regarding booking of patients to the surgical theatre combined with a surgeon from either the LA or PA team. In this way, we ensure that patients do not wait an unnecessarily long time for operation, after they are included. 2) The higher chance (66%) of being allocated to gait analysis in the first three blocks will enable us to verify the sample size for GDI. If needed, an adjusted allocation to gait analysis will be possible in the following block.

We have chosen to randomise the patients to a specific approach that automatically includes a specific team of surgeons with specialised skills in that particular approach, thus avoiding bias due to a learning curve. To avoid the risk of comparing surgeon skills and/or preference rather than the surgical procedure, a team of three experienced surgeons has been included in each group.

The randomisation code will be revealed to the investigators after the data analysis has been finalised. The primary outcome and supplementary PROM outcome will be blinded to the data collectors and data assessor (SR) throughout the trial, since all questionnaires will be answered at the patient’s home. The data collectors are also blinded to intervention when collecting data regarding the physical test. However, some difficulties regarding blinding must be mentioned. For practical reasons, it is not possible to maintain the blinding to intervention for the data collector (DBN) regarding the 3D gait analysis at three and twelve months’ follow-up, when placing the EMG electrodes in close relation to the scar at the hip. However, this will only influence a subgroup of the trial population and only the collection of explorative outcomes. Also, we will blind the patients by not telling them the given intervention, and prior to intervention we will explain to them why it is important that they are retained from this information. Nevertheless, there is a risk that the patient will guess the intervention from the name of the surgeon.

### Conclusion

The present trial will provide evidence for the choice of approach in the future, with focus on the patients’ perspective. The results of the trial will be submitted to peer-reviewed journals for publication, irrespective of the outcome, in accordance with the CONSORT guidelines for reporting of clinical trials and registration in ClinicalTrails.gov[84].

## Authors’ original submitted files for images

Below are the links to the authors’ original submitted files for images. Authors’ original file for figure 1

## Abbreviations

OA: Osteoarthritis
THR: Total hip replacement
PA: Posterior approach
LA: Lateral approach
PROM: Patient-reported outcome measures
RCT: Randomised controlled trial
30s CST: 30 second chair-stand-test
CE-angle: Center-edge-angle
NRS: Numeric Ranking Scale
HOOS: Hip Disability and Osteoarthritis Outcome Score
EQ-5D: EuroQol
UCLA: University of California Los Angeles activity score
HHS: Harris Hip Score
20WT: 20 meter walk test
TUG: Timed Up and Go
30s knee bend: 30 sec repeated unilateral knee bending test
ROM: Range of motion
GDI: Gait Deviation Index
MVC: Isometric maximal voluntary muscle contraction
WOMAC: Western Ontario and McMaster Universities Osteoarthritis Index
OHS: Oxford Hip Score.
