# Supplementary figures and images for: The effect of posterior and lateral approach on patient-reported outcome measures and physical function in patients with osteoarthritis, undergoing total hip replacement: a randomised controlled trial protocol
Source: BMC Musculoskelet Disord. 2014 Oct 27;15:354. doi: 10.1186/1471-2474-15-354 (PMC4216831; doi:10.1186/1471-2474-15-354)

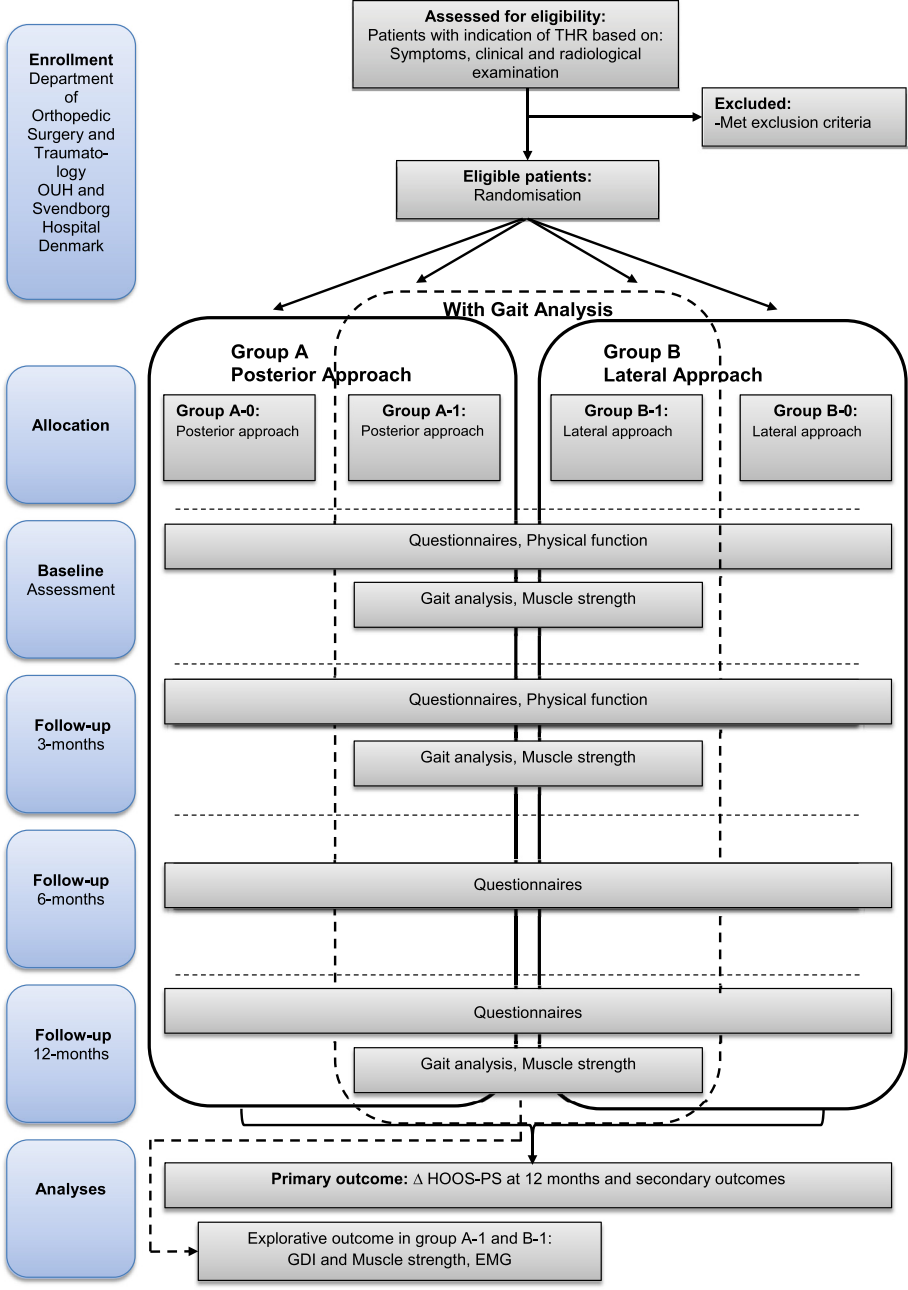

Supplement: Supplementary file 1 — Authors’ original file for figure 1 [file 12891_2013_2290_MOESM1_ESM.pdf]
